# Supplementary material for: Positive Childhood Experiences Associate with Adult Flourishing Amidst Adversity: A Cross Sectional Survey Study with a National Sample of Young Adults
Source: Int J Environ Res Public Health. 2022 Nov 13;19(22):14956. doi: 10.3390/ijerph192214956 (PMC9690672; doi:10.3390/ijerph192214956)
Supplement: Supplementary file 1 [file ijerph-19-14956-s001.zip › ijerph-1966904-supplementary.pdf]

Supplementary Materials

Table S1. Comparisons on the respondent characteristics between sample included for analysis and sample excluded due to missing data.

Participant Characteristics	Included Sample (*N* = 9468)	Excluded Sample (*n* = 1161)	*P*-Value for between Sample Comparison
**Age (in years)**			
Range	18–35	18–35	0.438
Mean (SD)	20.05 (1.67)	20.09 (1.93)	
**Gender, n (%)**			
Female	7129 (75.3)	686 (59.1)	
Male	2244 (23.7)	388 (33.4)	<0.001
Missing	95 (1.0)	87 (7.5)	
**Year in university, n (%)**			
Freshman	2146 (22.7)	239 (20.6)	
Sophomore	2652 (28.0)	374 (32.2)	
Junior	2986 (31.5)	327 (28.2)	<0.001
Senior	1342 (14.2)	145 (12.5)	
Graduate	259 (4.0)	13 (1.1)	
Missing	83 (0.9)	63 (5.4)	
**Marital status**			
Single	7554 (79.8)	746 (64.2)	
Married or cohabitate	107 (1.1)	17 (1.5)	<0.001
Other *	1807 (19.1)	398 (34.3)	

Note. Respondents who completed at least 75% of all survey measures were included in the final sample. Respondents with more than 25% non-response in any survey measures were excluded in the final sample. * Other includes missing, divorced, separated, widowed, or other marital status.

Table S2. Evaluations of psychometric properties of the Chinese version of Positive Childhood Experiences (C-PCEs) 9-item measure.

Internal consistency (*N* = 9468)	Cronbach's alpha = 0.724	
Test-retest reliability (1–2-month interval, *n* = 40)	Intraclass Correlation Coefficient (ICC) = 0.747<br>Weighted Kappa for each item ranges from 0.347 ("Feel able to talk to their families about feelings") to 0.637 ("Feel safe and protected by an adult in their home")	
Exploratory factor analysis (random half of total sample, *n* = 4734)	Principle axis factor analysis	Two Eigenvalues greater than 1.0.<br>Factor 1: 3.005 explains 33.38% of the variance.<br>Factor 2: 1.329 explains 14.76 % variance.<br>Two factors explain cumulative 48.15% variance.<br>See Figure S1 for Scree plot.<br>See Figure S2 for factor plot in rotated factor space, which supports 2 factor structure.
	Factor loading across items	0.54 ("Feel treated fairly at school") to 0.84 ("Feel their family stood by them during difficult times")
Confirmatory factor analysis (random half of total sample, *n* = 4734)	Indices of 2 factor model	CFI: 0.945<br>TLI: 0.924<br>SRMR: 0.027<br>RMSA 90% CI: 0.049

Note. CFI: Comparative Fit index; TLI: Tucker Lewis Index; SRMR: standardized root mean square residual; RMSA: root mean square error of approximation.

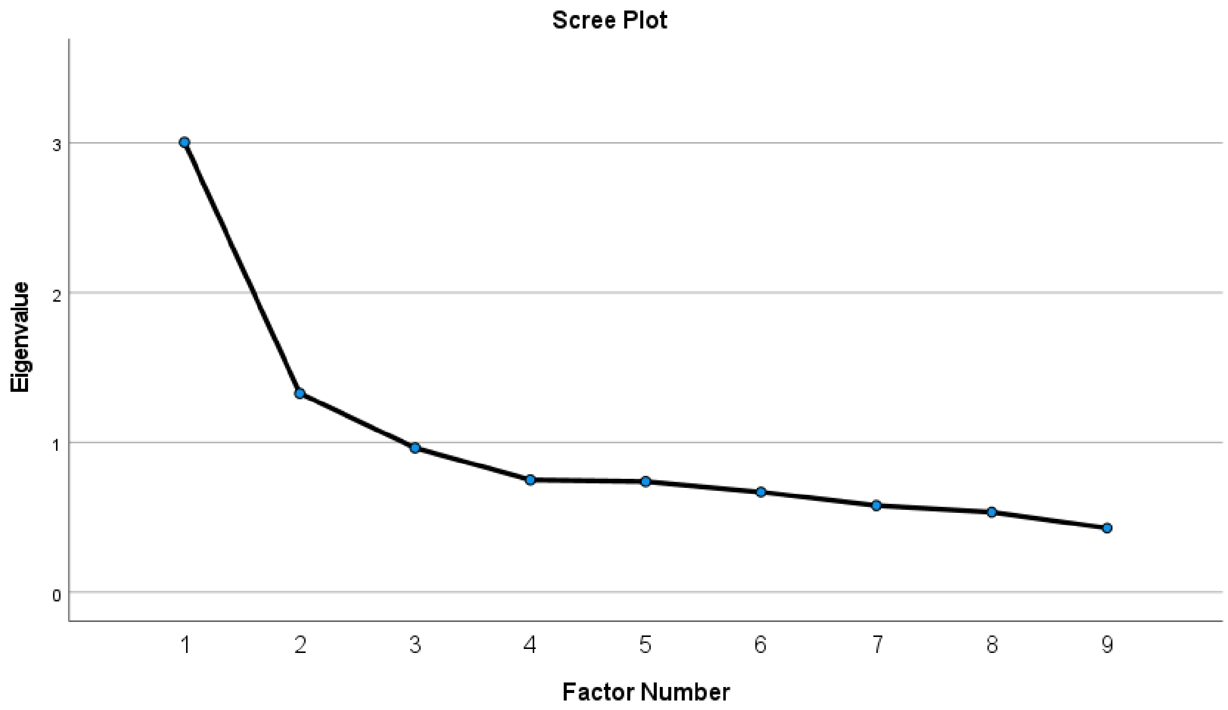

Figure S1. Scree plot for the Chinese version of Positive Childhood Experiences (C-PCEs) 9-item measure.

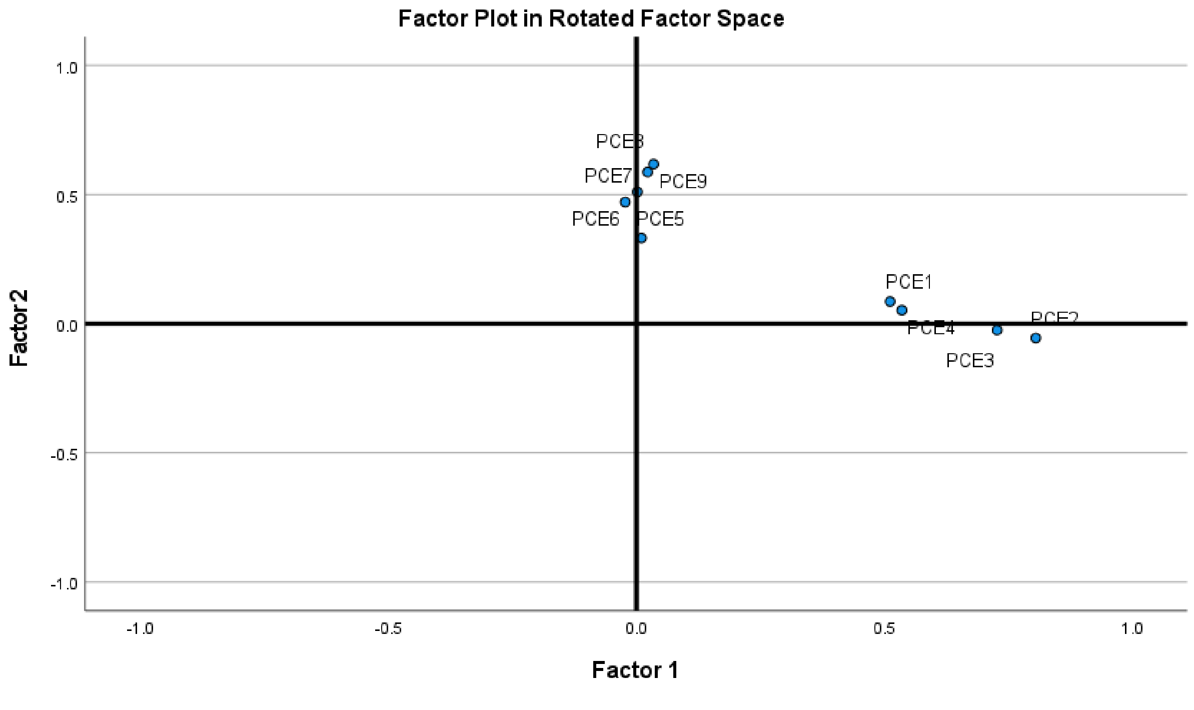

Figure S2. Factor plot in rotated factor space using Promax rotation for the Chinese version of Positive Childhood Experiences (C-PCEs) 9-item measure.
